# Poly(*o*-methoxyaniline) Chain Degradation Based on a Heat Treatment (HT) Process: Combined Experimental and Theoretical Evaluation

**DOI:** 10.3390/molecules27123693

**Published:** 2022-06-08

**Authors:** Jéssica Montenegro Santana da Silva, Adriano de Souza Carolino, Lilian Rodrigues de Oliveira, Douglas de Souza Gonçalves, Matheus Moraes Biondo, Pedro Henrique Campelo, Jaqueline de Araújo Bezerra, Ştefan Ţălu, Henrique Duarte da Fonseca Filho, Hidembergue Ordozgoith da Frota, Edgar Aparecido Sanches

**Affiliations:** 1 Laboratory of Nanostructured Polymers (NANOPOL-@nanopol_ufam), Federal University of Amazonas (UFAM), Manaus 69067-005, AM, Brazil; jessicamontenegro17@coppe.ufrj.br (J.M.S.d.S.); adriano.asc.fis@gmail.com (A.d.S.C.); lilianoliveira.r@gmail.com (L.R.d.O.); matheusbiondo@ufam.edu.br (M.M.B.); sanchesufam@ufam.edu.br (E.A.S.); 2 Graduate Program in Materials Science and Engineering (PPGCEM), Faculty of Technology, Federal University of Amazonas (UFAM), Manaus 69067-005, AM, Brazil; hdffilho@ufam.edu.br (H.D.d.F.F.); hfrota@ufam.edu.br (H.O.d.F.); 3 Graduate Program in Physics (PPGFIS), Federal University of Amazonas (UFAM), Manaus 69067-005, AM, Brazil; douglas.ufam@gmail.com; 4 Department of Food Technology, Federal University of Viçosa (UFV), Viçosa 36570-900, MG, Brazil; pcampelo.felix@gmail.com; 5 Analytical Center, Federal Institute of Education, Science and Technology of Amazonas (IFAM), Manaus 69020-120, AM, Brazil; jaqueline.araujo@ifam.edu.br; 6 The Directorate of Research, Development and Innovation Management (DMCDI), Technical University of Cluj-Napoca, 15 Constantin Daicoviciu St., 400020 Cluj-Napoca, Romania; 7 Laboratory of Synthesis of Nanomaterials and Nanoscopy (LSNN), Federal University of Amazonas (UFAM), Manaus 69067-005, AM, Brazil

**Keywords:** heat treatment, poly(*o*-methoxyaniline), XRD, Le Bail method

## Abstract

Poly(*o*-methoxyaniline) emeraldine-salt form (ES-POMA) was chemically synthesized using hydrochloric acid and subjected to a heat treatment (HT) process for 1 h at 100 °C (TT_100_) and 200 °C (TT_200_). The HT process promoted a progressive decrease in crystallinity. The Le Bail method revealed a decomposition from tetrameric to trimeric-folded chains after the HT process. The unheated POMA-ES presented a globular vesicular morphology with varied micrometric sizes. The heat treatment promoted a reduction in these globular structures, increasing the non-crystalline phase. The boundary length (S) and connectivity/Euler feature (χ) parameters were calculated from the SEM images, revealing that ES-POMA presented a wide distribution of heights. The TT_100_ and TT_200_ presented a narrow boundary distribution, suggesting smoother surfaces with smaller height variations. The UV-VIS analysis revealed that the transition at 343 nm (nonlocal *π* → π*) was more intense in the TT_200_ due to the electronic delocalization, which resulted from the reduced polymer chain caused by the HT process. In addition to the loss of conjugation, counter ion withdrawal reduced the ion-chain interaction, decreasing the local electron density. This result shows the influence of the chlorine counter ions on the peaks position related to the HOMO → LUMO transition, since the π → polaron transition occurs due to the creation of the energy states due to the presence of counter ions. Finally, the electrical conductivity decreased after the HT process from 1.4 × 10^−4^ S.cm^−1^ to 2.4 × 10^−6^ S.cm^−1^ as result of the polymer deprotonation/degradation. Thus, this paper proposed a systematic evaluation of the POMA molecular structure and crystallite size and shape after heat treatment.

## 1. Introduction

Intrinsically conducting polymers (ICPs) have attracted attention due to their potential for the development of new technological applications. Their properties are dependent on the manufacturing process and crystallinity [1,2]. For this reason, some physicochemical properties are directly related to their crystal structure including lattice parameters and crystallite size, as well as crystal imperfections resulting in important effects on their performance [3].

New technological applications including polyaniline (PANI) have become advantageous due to their high monomer availability and simple polymerization methodology, as well as their high stability and electrical conductivity at room temperature. However, the emeraldine-salt form (ES-PANI) is difficult to solubilize, limiting its potential for new technological and commercial applications. For this reason, various PANI derivatives have been investigated [4]. Among them, the poly(*o*-methoxyaniline) emeraldine-salt form (ES-POMA) [5,6] presents a molecular structure similar to that of PANI, besides showing an improved solubility due to the *o*-methoxy substitution.

The thermal stability of ES-POMA represents a significant parameter for commercial applications due to the influence of temperature on its oxidation state and molecular structure. There is a lack of reports focusing on the changes of the structural, electrical, and morphological properties of POMA after heat treatment (HT) [7]. For this reason, the present study evaluated the crystal structure of POMA maintained at 100 °C (TT_100_) and 200 °C (TT_200_) in order to provide a systematic evaluation of the crystalline phase modification. The structural, spectroscopic, and morphological aspects of heat-treated semi-crystalline materials continue to be an interesting topic of research [8,9,10,11,12,13].

Understanding the modification of the regular arrangement of polymer chains is important for the prediction of processing methods, new properties, and applications. The X-ray diffraction technique (XRD) was applied in order to examine the long-range order achieved as a consequence of very short-range interactions. The XRD measurements were also used as input data for the Le Bail method, as well as to estimate the percentage of crystallinity. The Le Bail method was performed using the Fullprof program [14] to obtain the unit cell parameters. The line-broadening analysis of the XRD patterns allowed a real description of the microstructural features of the treated and untreated POMA. The linear combination of spherical harmonics was applied to evaluate the anisotropic crystallite size and shape. The scanning electron microscopy (SEM) technique was applied to evaluate the influence of the heat treatment on the powder polymer morphology. Density functional theory [15,16] was employed to investigate the polymer geometry optimization and frequency calculation. The absorption spectra were computed using time-dependent DFT [17] to verify the electronic transitions experimentally observed. Furthermore, the binding energies and binding free energies of the dimers, trimers, and tetramers were also calculated. Then, these results were correlated with the DC electrical conductivity measurements.

## 2. Results

### 2.1. XRD Analysis and Percentage of Crystallinity

POMA powder was maintained at 100 °C and 200 °C for 1 h to verify the influence of the HT process on its crystal structure. Consistent with the scientific literature [18,19]. the untreated ES-POMA presented a higher crystalline XRD pattern, with well-defined peaks at 2θ = 7.8°; 13.0°; 18.0°, and 24.8°. In contrast, after the HT process, structural degradation was observed: the peaks located at 2θ = 7.8°; 13.0°, and 18.0° presented lower intensities after heat treatment at 100 °C (TT_100_) for 1 h. Moreover, the polymer treated at 200 °C (TT_200_) lost the peaks previously observed in the unheated sample, and only one well-defined peak was observed at 2θ = 24.8°.

### 2.2. Le Bail Method

The lattice parameters’ modification was performed in order to accommodate both the *o*-methoxy groups and the chlorine counter ions (in the case of the untreated polymer) present in the POMA structure. For this reason, the unit cell volume was increased (when compared to that of polyaniline unit cell values). A good fit was obtained by introducing new values of the “*a*” and “*b*” unit cell parameters around 7.10275 Å and 11.39338 Å, respectively. Moreover, after the HT process, the “*c*” unit cell value was decreased from 18.82489 Å to 15.62562 Å. The value of 18.82489 Å is similar to the length of an aniline tetramer [20], excluding the end-capped structure. Supposing the chains lie along this unit cell direction, we suggest that the HT process allows the chains’ decomposition from tetrameric to trimeric-folded chains after treatment at 200 °C.

### 2.3. Experimental UV-VIS Analysis

The UV-VIS spectrum of ES-POMA presented well-defined absorption peaks at 272 nm, 343 nm, 433 nm, 526 nm, and 810 nm. On the other hand, the spectrum of TT_200_ presented peaks at 272 nm, 343 nm, 428 nm, 526 nm, and 780 nm. The absorptions at 272 nm and 343 nm were assigned both to the *π* → π* aromatic rings and non-local transitions. The absorptions at 428 nm and 526 nm were assigned to the polaron → π* and *n* → π* transitions of the polymer quinoid structure. A strong absorption was observed in both samples in the region of 720 and 810 nm, which was attributed to the *π* → *polaron* transition.

### 2.4. SEM Analysis

The untreated ES-POMA clearly presented a globular vesicular morphology with different micrometric diameters. These globular particles present an internal composition of crystalline and non-crystalline phases. The increase in temperature promoted the decrease in the globular morphology, as well as their globular vesicular behavior. After the HT process, these structures seemed to increase the non-crystalline contribution. These data agree with the XRD analysis, which showed increasingly non-crystalline diffraction patterns when the temperature was increased.

### 2.5. Geometric Optimization

The binding energy between the tetramer chain and counter ions was found to be around −88.88 kcal.mol^−1^. During the HT process, this energy was required to initiate the release of the chloride counter ions from the polymeric structure, representing the first stage of degradation. Then, the resulting tetrameric structure presented a binding energy value between rings I and II of approximately −186.37 kcal.mol^−1^. When this energy value was applied to the system, the polymer chain was converted to a trimeric form (representing the second stage of the HT process), and the interaction of the counter ions with the polymer chain was reduced. For this reason, this system showed a positive binding/free energy between the trimeric chain and the counter ions, showing that this reaction did not occur spontaneously. We also calculated the undoped forms of the tetramer and trimer as a method of comparing their respective UV-VIS absorption spectra, showing the influence of the counter ions on the structure, corroborating the experimental results.

### 2.6. Theoretical UV-VIS Analysis

Absorptions resulted from the *π* → π* transition, predicted by the calculated spectrum as the transition *H* − 1 → *L +* 2 (60%) (undoped-POMA trimer), *HOMO* → *L +* 2 (71%) (doped-POMA trimer), *HOMO* → *L +* 18 (27%) (undoped-POMA tetramer), and *HOMO* → *L +* 2 (83%) (doped-POMA tetramer). The transition resulting from the *π*–polaron transition was predicted as *HOMO* → *LUMO* (46%) (doped-POMA trimer) and *HOMO* → *LUMO* (50%) (doped-POMA tetramer).

### 2.7. Electrical Conductivity

The electrical conductivity value of the untreated ES-POMA was found to be around 1.4 × 10^−4^ S.cm^−1^. However, a decrease in electrical conductivity was observed after the HT process, as expected, from 7.2 × 10^−5^ S.cm^−1^(TT_100_) to 2.4 × 10^−6^ S.cm^−1^ (TT_200_).

## 3. Discussion

### 3.1. XRD Analysis

**Figure 1:**
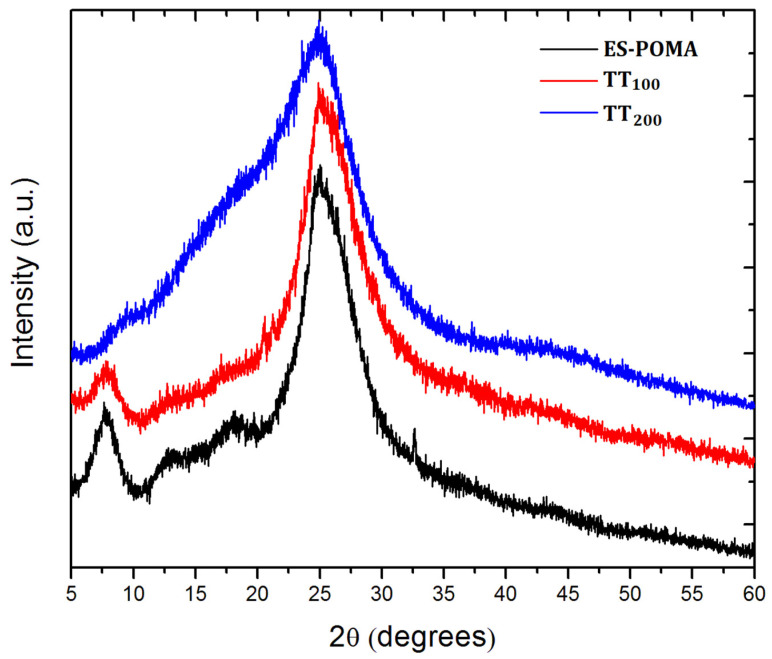
XRD patterns of the untreated (ES-POMA) and treated POMA at 100 °C (TT_100_) and 200 °C (TT_200_).

To the best of our knowledge, thermal treatment of POMA has been reported in the scientific literature using only differential scanning calorimetry (DSC) to investigate entrapped water in the powder form of POMA [19]. Figure 1 shows the XRD patterns of the untreated and treated ES-POMA.

The XRD patterns clearly evidenced the loss of crystallinity as a function of temperature, as expected. The patterns obtained after the HT process presented a smaller number of peaks, which were broader when compared to those of as-synthesized ES-POMA. This result is related to the increase in the non-crystalline contribution and the decrease in the crystallite size. Moreover, the decreased crystallinity of the treated polymers is also due to the polymer deprotonation [21] and degradation.

Many methods have been applied to estimate the percentage of crystallinity of semi-crystalline materials, considering a two-phase model of crystallites embedded in a non-crystalline matrix. However, these methods do not always provide similar results. The deconvolution method [22,23] is the most used to access the crystalline phase of a semi-crystalline material from a XRD pattern. An important hypothesis for this analysis is that the non-crystalline contribution is the main contributor to peak broadening. However, in addition to the non-crystalline content, there are other intrinsic factors.

**Figure 2:**
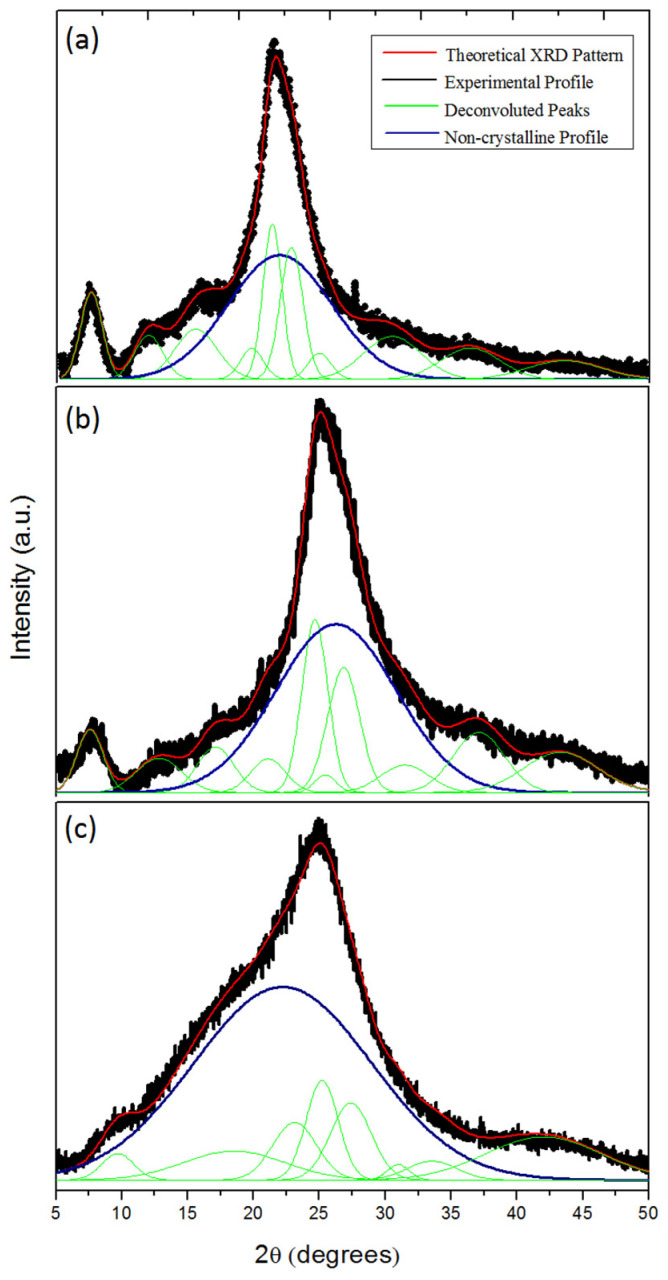
Peak deconvolution of the XRD patterns of the (**a**) untreated and treated POMA at (**b**) 100 °C and (**c**) 200 °C.

The peak deconvolution of the XRD patterns of the untreated and treated POMA are shown in Figure 2. The Chebyshev polynomial was subtracted from the total pattern, allowing the crystalline peaks’ assessment. Peaks were best fitted using Gaussian curves. Comparing the experimental area with those of polynomial curves, the percentage of crystallinity decreased from (59 ± 2)% for the untreated ES-POMA. However, the TT_100_ and TT_200_ samples presented values of (53 ± 2)% and (36 ± 2)%, respectively.

Table 1 shows the refined parameters of POMA-ES, TT_100_, and TT_200_ polymers. Structural refinement of untreated and treated POMA was performed using the initial parameters reported for the end-capped tetramer of aniline [20].

### 3.2. Le Bail Method

The Le Bail method [24] has recently been performed to obtain the structural information of semi-crystalline materials [11]. This process iteratively uses the Rietveld decomposition formula for whole powder pattern decomposition (WPPD) through the Fullprof program package [14]. The size of the coherent diffraction domains is based on the integral breadth of line profiles, from which the apparent size can be obtained [3]. Anisotropic size broadening can be written as a linear combination of spherical harmonics (SHP), and it is supposed that the anisotropic size contributes only to the Lorentzian component of the total Voigt function. Figure 2 shows the peak deconvolution of the XRD patterns of the untreated and treated POMA.

**Figure 3:**
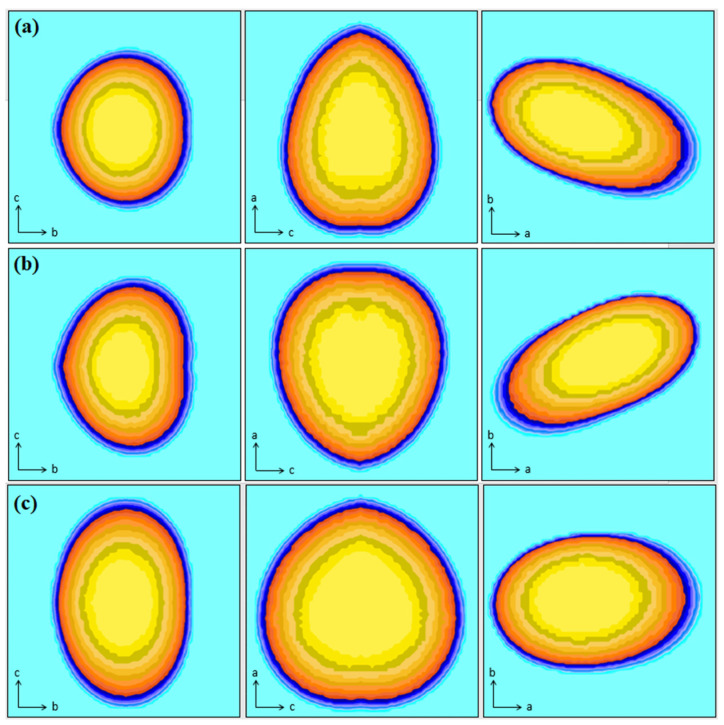
Crystallite shape from the Le Bail refinement of (**a**) ES-POMA, (**b**) TT_100_, and (**c**) TT_200_ samples along the directions [100], [010], [001].

Figure 3 shows the crystallite shape generated after the refinement of ES-POMA, TT_100_, and TT_200_ samples along the directions [100], [010], [001]. As observed in the XRD patterns, the increase in the non-crystalline regions due to the HT process influenced the crystallite average size and shape. A decrease of approximately 30%, from 32 Å (untreated form) to 22 Å (TT_200_), was observed. A prolate crystallite shape was observed in the untreated ES-POMA. After treatment at 200 °C, the crystallite size anisotropy was increased, resulting in more globular crystallites.

### 3.3. Experimental UV-VIS Analysis

**Figure 4:**
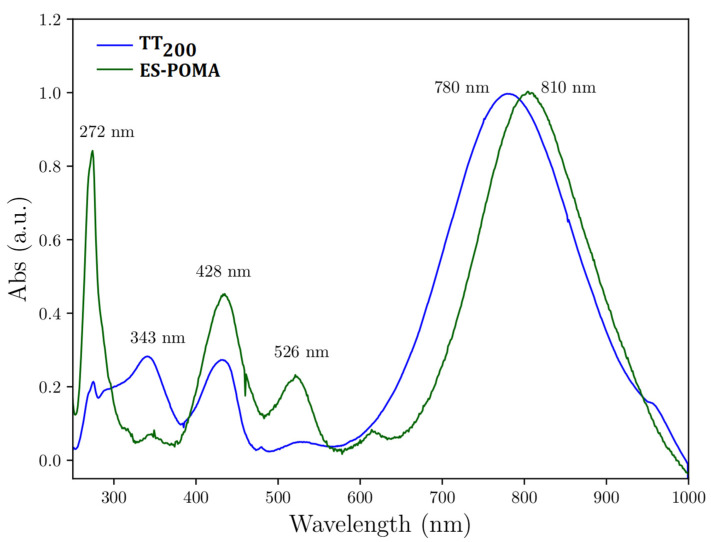
UV-VIS spectra of ES-POMA and TT_200_.

Figure 4 shows the UV-VIS spectra of the ES-POMA and TT_200_ samples. The electronic transitions were almost similar. However, the transition at 343 nm (nonlocal *π* → π*) was more intense in the TT_200_ sample when compared to the untreated ES-POMA. These transitions occurred due to the electronic delocalization resulting from the reduced polymer chain caused by the HT process. These results were also verified by the Le Bail refinement, showing the resulting trimeric polymer chain in TT_100_ and TT_200_. Consequently, the HT process reduced the structural defects formed by the interaction of counter ions still present in the polymer structure.

In addition to the loss of conjugation, the removal of counter ions (caused by the increase in temperature) reduced the ion-chain interaction, decreasing the local electron density. Therefore, a blue shift was observed at the most intense peak (from 810 nm to 780 nm), which was related to the *π* → polaron transition, indicating that a higher energy was necessary for this electronic transition, since the reduction in structural defects modifies the polaron state energy levels, increasing the system gap energy.

### 3.4. SEM Analysis

**Figure 5:**
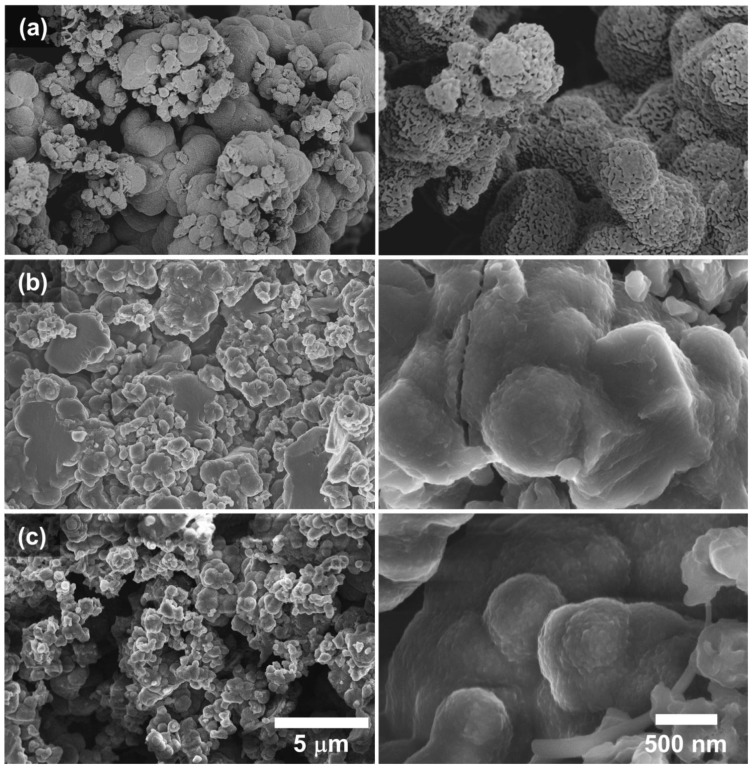
SEM micrographs of the (**a**) untreated and treated samples, (**b**) TT_100_, and (**c**) TT_200_. The magnification is 15,000× and 100,000×.

To illustrate the modification of the ES-POMA micromorphology due to the HT process, SEM micrographs showing the surface topographies of the untreated and treated POMA are presented in Figure 5 (magnifications of 15,000× and 100,000×). Surface changes induced by the HT process were clearly observed, reducing the globular vesicular morphology.

A more in-depth analysis regarding the effects of the HT process on ES-POMA was performed. A set of SEM images with magnifications of 100,000× was considered. The images from Figure 5 were magnified at specific regions. Usually, the morphological attributes of surfaces are obtained through probe scanning techniques, such as AFM, STM, or even profilometry. These techniques allow the evaluation of texture parameters due to the third dimension, represented by the *z* axis. However, surface evaluation through SEM images is possible even considering two-dimensional parameters. Based on the variation in the intensity of the gray tones of each pixel (pixel intensity values 0–255), the image calibration (related to the current scale length) must be performed to obtain a precise reference of both the *x* and *y* dimensions in the image plane. In the present study, values from 0 to 255 were assigned to the blackest and whitest pixels, respectively, aiming to convert them to height values.

**Figure 6:**
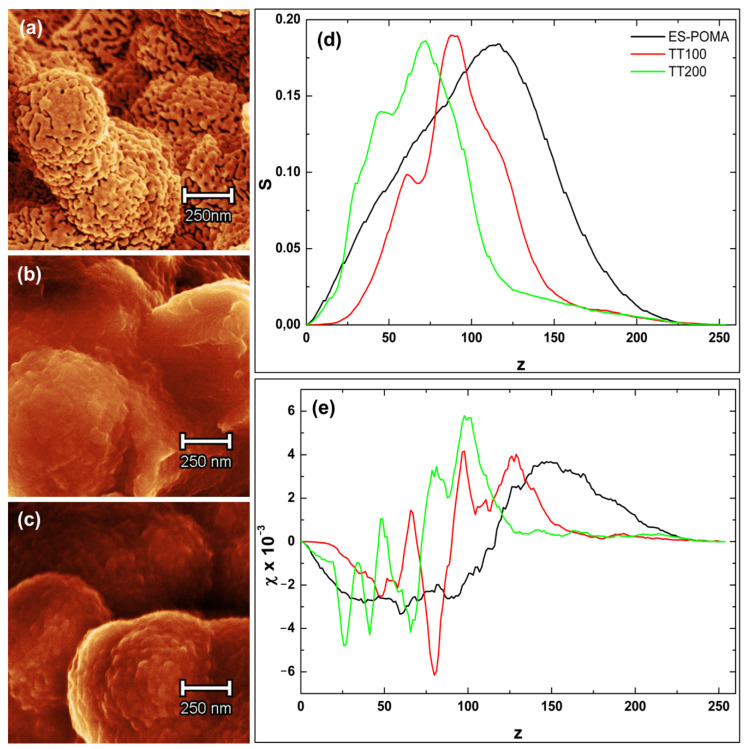
Two-dimensional zoom reconstruction of SEM images (100,000×) from (**a**) ES-POMA, (**b**) TT_100_, and (**c**) TT_200_. Representation of the (**d**) Minkowski surface and (**e**) Minkowski connectivity functions of images (**a**–**c**).

Figure 6a–c shows the 2D reconstructions of the aforementioned cutouts. Colors were used only to highlight micro and nanoscale differences between the samples.

The boundary length (S) and connectivity/Euler feature (χ) parameters were calculated according to the separation of SEM images into low and high parts according to the range of 0–255 based on a threshold. These parameters represent statistical data capable of characterizing the morphology and geometric structure of a system containing several objects irregularly distributed in 2D and 3D space. In addition, they have been extensively applied in such analyses due to their similarity to physically useful parameters [25]. Figure 6d,e shows, respectively, the Minkowski boundary and connectivity curves for three samples as a function of the threshold level (*z*). Figure 6d shows that ES-POMA presents a wide boundary distribution, indicating a possible greater distribution of heights, which was observed in the SEM images. The TT_100_ and TT_200_ curves were similar and exhibited a considerably reduced symmetrical behavior when compared to that of untreated ES-POMA. These curves presented a narrow boundary distribution, suggesting smoother surfaces with smaller height variations. The S(*z*) curves tended to zero for both *z* → 0 and *z* → 255, growing rapidly, then decreasing to a local minimum, and rapidly increasing again until reaching a maximum. Based on these data, POMA undergoes a complex morphological transformation due to the applied HT process.

The Minkowski connectivity (Figure 6e) enabled the description of the topological structure of the surface patterns, calculating the difference in the number of white and black level regions [26]. This is achieved using the radially Gaussian mean power spectral density function, a parameter which describes the connectivity of spatial patterns that are particularly sensitive to topological changes in the pattern (of a fractal nature). The ES-POMA connectivity presented a greater value. After the HT process, it decreased for the TT_200_ and TT_100_ samples. The χ connectivity can be positive, indicating surfaces presenting a higher density of peaks, or negative, indicating the predominance of lower regions or valleys. These variations in peak and valley values can be important for technological applications. These results are also in agreement with those observed in SEM micrographs [27].

**Figure 7:**
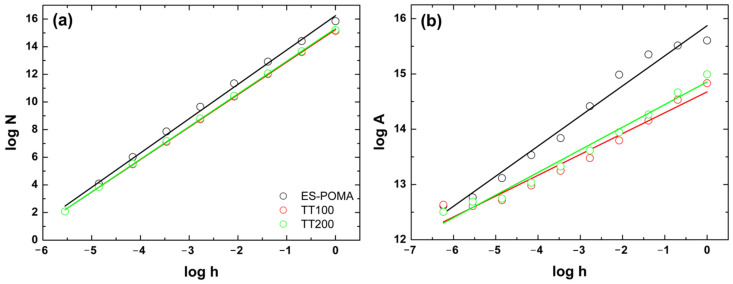
Fractal dimensions calculated as (**a**) cube counting and (**b**) triangulation.

Mandelbrot proposed the fractal theory to describe this self-similarity of natural features [28]. Several programs are available to calculate the fractal dimension through different methodologies from grayscale images [29]. Here, we also extracted the Df data from the Gwyddion program [30], and Figure 7 shows the graphs of the fractal dimension calculations and the respective adjustments for the analyzed samples.

The parameters associated with Df using the cube counting method were 2.48, 2.35, and 2.36 for ES-POMA, TT_100_, and TT_200_, respectively. The parameters associated with Df using the triangulation method were 2.54, 2.38, and 2.41 for ES-POMA, TT_100_, and TT_200_. These results complemented the qualitative analysis. A decrease in the values between ES-POMA and TT_100_ was observed. However, Df remained practically constant for TT_100_ and TT_200_. The fractal dimension values generally range from two to three and are directly related to the surface complexity. For smooth surfaces, the values will be closer to two and increase to three when the roughness is increased [31]. For this reason, the HT process influenced the Df values to approach two, indicating that the surfaces tended to become less rough. This evidence has already been pointed out in previous analyses.

### 3.5. Geometric Optimization

**Figure 8:**
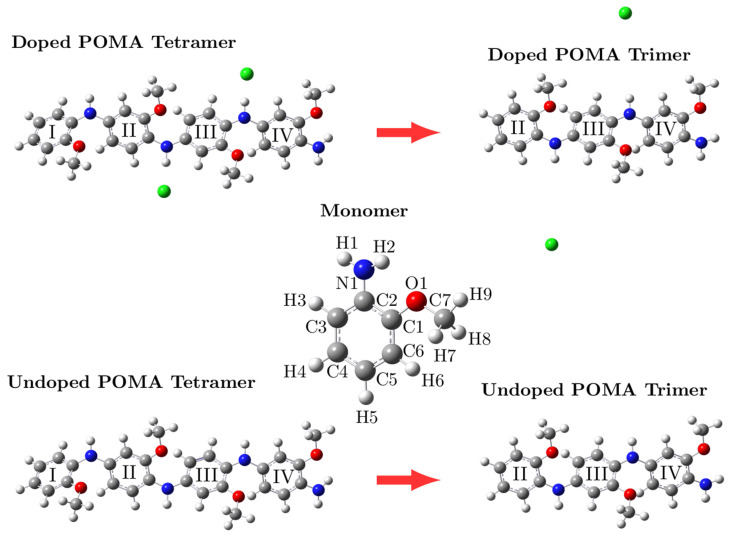
Geometric optimization of doped-POMA (ES-POMA) tetramer, undoped-POMA tetramer, doped-POMA (ES-POMA) trimer, and undoped-POMA trimer.

DFT calculations were performed to access the geometric optimization, density of states (DOS) projected by atoms, and UV-VIS spectra of untreated and treated ES-POMA. Four systems were considered: (i) doped-POMA (ES-POMA) tetramer, (ii) undoped-POMA tetramer, (iii) doped-POMA (ES-POMA) trimer, and (iv) undoped-POMA trimer. The geometric optimization of all systems is shown in Figure 8.

All systems presented linear geometry, with distortions between their ring constituents, as well as regular and similar bond lengths (as shown in Table 2). A slight variation in the C–C bond length was observed from 1.39 Å to 1.40 Å. The C–N and N–H bond lengths were found, respectively, in the range of 1.36–1.41 Å and 1.00–1.04 Å. The C–O bond length was 1.39 Å. The distances at which the chloride counter ions interacted with N–H were also accessed. In the tetrameric polymers, the counter ions interacted with the polymer chain at a distance of 1.95 Å, while in the trimeric polymer structure, the chloride counter ions did not show a significant interaction with the polymer chain, reaching 5.91 Å.

To evaluate the stability of the structures and the reaction mechanism induced by the HT process, the binding energy (Δ*E_binding_*) and the Gibbs free energy of binding (Δ*G_binding_*) were calculated, as shown in Table 3. The energy required to rupture a bond between one of the rings of the tetrameric system was considered, as well as the energy difference considering the presence of the chlorine counter ions. During the HT process, the binding energy between the tetramer chain and the counter ions (−88.88 kcal.mol^−1^) allowed the initiation of the release of counter ions in the first stage of degradation. As a result, the tetrameric structure presented a binding energy value between rings I and II of approximately −186.37 kcal.mol^−1^, where the polymer chain was converted to a trimeric form and at the same time, reduced the interaction of the counter ions with the polymeric chain. For this reason, this system presented a positive binding/free energy between the polymer chain and counter ions, showing that this reaction did not occur spontaneously. This configuration represented the last stage of the HT process.

### 3.6. Theoretical UV-VIS Analysis

**Figure 9:**
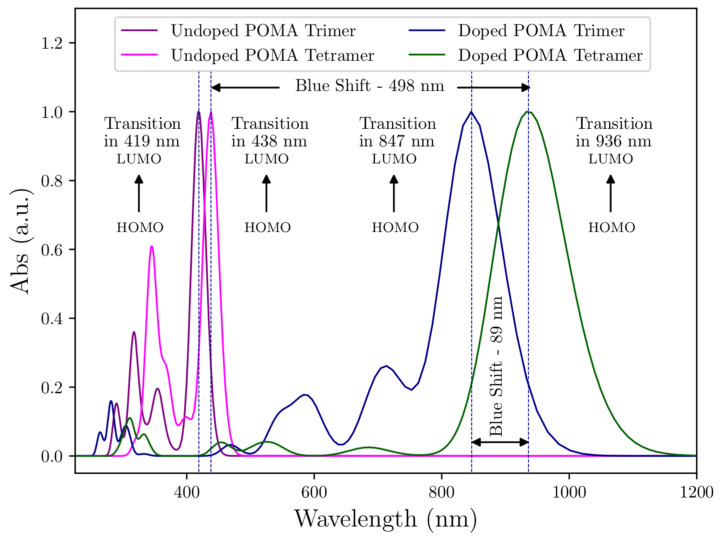
Theoretical UV-VIS spectra of doped-POMA (ES-POMA) tetramer, undoped-POMA tetramer, doped-POMA (ES-POMA) trimer and undoped-POMA trimer.

To investigate the energy absorptions observed in the experimental UV-VIS spectra, calculation of the energy transitions by the TD-DFT method was performed. The experimental (Figure 4) and theoretical (Figure 9) spectra presented a satisfactory similarity. Table 4 shows the comparison of wavelengths and electronic transitions between the experimental and theoretical systems.

Figure 9 shows that the HOMO → LUMO transitions of all systems presented a blue shift. For the undoped polymers, the blue shift was observed due to the reduced amount of conjugation. In regards to the effect of the HT process on ES-POMA, a HOMO → LUMO transition was observed at higher wavelengths when the chlorine counter ions were present in the as-synthesized polymer chain structure. Then, during the HT process, the chlorine counter ions were partially withdrawn from 100 °C to 200 °C, reducing their interaction with the polymer chains. This result shows the influence of the chlorine counter ions on the peaks’ position relative to the HOMO → LUMO transition, since the *π* → polaron transition occurs due to the creation of the energy states due to the presence of the counter ions.

**Table 4:** Wavelengths and electronic transitions of experimental and theoretical systems.

Experimental		Theoretical							
Trimer	Tetramer Cl2	Trimer		Trimer Cl2		Tetramer		Tetramer Cl2	
UV-VIS (nm)		UV-VIS (nm)	Trans.	UV-VIS (nm)	Trans.	UV-VIS (nm)	Trans.	UV-VIS (nm)	Trans.
272	272	290	H − 1 > L + 2 (60%)	280	HOMO > L + 2 (71%)	317	HOMO > L + 18 (27%)	310	HOMO > L+2 (83%)
343	343	317	HOMO > L + 11 (43%)	304	HOMO > L + 1 (77%)	345	HOMO > L + 12 (37%)	334	HOMO > L + 1 (83%)
428	433	354	HOMO > L + 5 (60%)	464	H − 10 > LUMO (74%)	369	HOMO > L + 4 (70%)	452	H − 11 > LUMO (85%)
526	526	419	HOMO > LUMO (90%)	586	H − 8 > LUMO (84%)	438	HOMO > LUMO (87%)	525	H − 3 > LUMO (80%)
780	810	-		710	H − 1 > LUMO (63%)	-		684	H − 1 > LUMO (70%)
-	-	-		847	HOMO > LUMO (46%)	-		936	HOMO > LUMO (50%)

#### HOMO-LUMO Analysis

**Figure 10:**
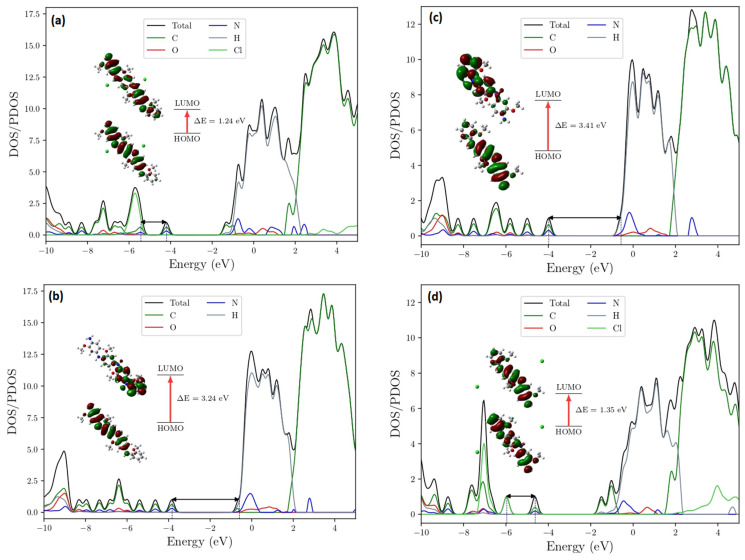
Gap energy and density of states (DOS/PDOS) of the (**a**) doped-POMA tetramer, (**b**) undoped-POMA tetramer, (**c**) undoped-POMA trimer, and (**d**) doped-POMA trimer.

Figure 10a–d shows the gap energy and density of states (DOS/PDOS) of the doped-POMA tetramer, undoped-POMA tetramer, undoped-POMA trimer, and doped-POMA trimer. The chlorine counter ions created energy levels below the HOMO orbital when compared to the undoped tetramer. Thus, the first unoccupied LUMO state in the doped tetramer (~−4.19 eV) became the first occupied HOMO state (~−3.83 eV) in the undoped tetramer. When compared with the undoped tetramer, the system changed from a conductor (gap = 1.24 eV) to an insulator (gap = 3.24 eV). We see that the energy gaps of the undoped tetramer and trimer increased the gap to 3.41 eV. Similarly, when comparing the doped systems, the gap increased from 1.24 to 1.35. Thus, the counter ions (even with a low interaction with the trimer chain) assigned the system energy levels between the HOMO and LUMO states when compared to the undoped systems, but with a wider energy gap.

### 3.7. DC Electrical Conductivity

Several parameters influence on the mobility of counter ions along the polymer chains in ICP materials. These factors are mainly related to the (i) synthesis methodology [32,33,34], (ii) regular packing of the polymer chains (or polymer crystallinity), (iii) ring side group at the *ortho*, *meta*, or *para* positions [8,35,36,37], and (iv) the nature of the doping acid and counter ion size [38], as well as (v) the possible chemical and/or physical interactions between counter ions and ring side groups [39].

The electrical conductivity value of the untreated ES-POMA was found to be 1.4 × 10^−4^ S.cm^−1^, which is similar to that found in the scientific literature [40,41]. However, a decrease in electrical conductivity was observed after the HT process, as expected, from 7.2 × 10^−5^ S/cm (TT_100_) to 2.4 × 10^−6^ S.cm^−1^ (TT_200_). These results agree with those found via the XRD technique, Le Bail refinement, and theoretical calculations, showing that the counter ion withdrawal, polymer chain degradation, and loss of crystallinity were a consequence of the HT process.

## 4. Methods and Materials

### 4.1. Synthesis of ES-POMA and Heat Treatment (HT)

Poly(*o*-methoxyaniline) emeraldine-salt form (ES-POMA) was synthesized based on the method described elsewhere [18]. Solution I was prepared by dissolving 28 mL of distilled *o*-anisidine monomer in HCl 1 M (500 mL) at 25 °C. Solution II was obtained by adding a stoichiometrically calculated amount of ammonium persulfate (APS) to HCl 1 M (200 mL). Solution II was added drop-by-drop to Solution I under constant stirring for 3 h. The dark dispersion was vacuum filtered and washed using acetone. Then, ES-POMA (5 g) was maintained for 1 h in a tube oven at 100 °C (TT_100_) and 200 °C (TT_200_). Temperature programming was performed using the Flycon software.

### 4.2. XRD Analysis and Percentage of Crystallinity

XRD data were obtained on a Rigaku Rotaflex diffractometer equipped with a graphite monochromator and rotating anode tube, operating with CuK_α_, 40 kV, and 40 mA. Powder diffraction patterns were obtained in the range 2θ = 5–60°, step of 0.02°, and 5 s/step. The peak fitting module program [42,43] was used for the peak decomposition of the semi-crystalline patterns. The ratio between the sums of the peak areas to the area of the non-crystalline broad halo due to the non-crystalline contribution was applied to assess the crystalline phase percentage.

### 4.3. Le Bail Whole Powder Pattern Decomposition Method

The Le Bail method was performed to assess the cell parameters, and crystallite size and shape from the XRD patterns. The software package Fullprof [14] was used to perform the method. All parameters were refined by the least-squares method [44]. The *pseudo*-Voigt function modified by Thompson–Cox–Hastings was used as peak profile function [45]. Instrumental resolution function parameters were obtained from a LaB_6_ standard. The crystal parameters of the end-capped tetramer of aniline [20] were used as initial parameters (triclinic P–1; *a* = 5.7328 Å; *b* = 8.8866 Å; *c* = 22.6889 Å; *α* = 82.7481°; *β* = 84.5281°, and *γ* = 88.4739°). Linear combinations of spherical harmonics (SHP) [46] were used to evaluate the crystallite anisotropy.

### 4.4. Ultraviolet-Visible (UV-VIS) Spectroscopy

Absorbance measurements were performed on a Biotek Epoch 2 spectrophotometer using a quartz cuvette (optical path of 1 cm) from 200 cm^−1^ to 1000 cm^−1^.

### 4.5. SEM Analysis

SEM experiments were performed using a Supra 35, Carl Zeiss, 1.0 kV. Powder samples were deposited on a carbon tape, and the surface morphology was obtained at 25 °C. The software Gwyddion 2.59 [30] was used to perform the morphological analysis from SEM data. As morphological descriptors, additional nanoscale information was extracted from two Minkovski Functionals (MF): boundaries distributions (S) and connectivity (χ: the Euler–Poincare characteristic), in addition to the fractal dimension (Df).

MF parameters provide information about variations in surface morphology following a deterministic but stochastic behavior on local geometrical and morphological structures [25]. They can be expressed as (i) S_(z)_ = N_bond_/N and (ii) χ_(z)_ = (C_white_ − C_black_)/N. The parameter *N* is the total number of pixels; *N_bond_* is the number of white-black pixel boundaries, and *C_white_* and *C_black_* are, respectively, the number of continuous sets of white and black pixels.

The fractal dimension calculations were based both on cube counting and triangulation. In the first case—derived directly from a definition of the box-counting fractal dimension—the surface is divided into a number *N* of square spots, where each one corresponds to a set of pixels in the image, and later, the height *h* of each spot is evaluated. In this case, the values of Df are obtained through the slope of the graph of log(*N*) versus log(*h*) [30]. The second method is also based directly on the fractal dimension of box-counting, being similar to the previous method. However, this method considers a grid of triangles of side length (*L*) which is placed over the image. The areas of all the triangles are calculated and summed to obtain an approximation of the surface area S(*L*) corresponding to *L*. The process is continuous, such that the grid size is decreased by a successive factor of 2 until *l* corresponds to the distance between two adjacent pixel points [47].

### 4.6. Theoretical and Computational Methods

Geometry optimization and frequency calculations were performed using the density functional theory [15] implemented in the Gaussian 03 package [48]. The DFT calculations considered the hybrid exchange-correlation functional B3LYP [49] with the aug-cc-pVDZ augmented double-zeta basis set [50]. This methodology was used due to its good representation of the molecular orbital and spectroscopic property calculations [51,52,53]. The ground-state geometries were obtained with an RMS force convergence criterion of 3 × 10^−4^ au (default value in Gaussian) and with an “ultrafine” integration grid. All geometries represented the local minima of the potential energy surface, i.e., presenting only positive frequencies. The absorption spectra were calculated using time-dependent DFT [54] with the same exchange-correlation functional and basis set. The polarizable continuum model (PCM) was employed to simulate the solvent effect in calculation of the UV-VIS spectra [53]. Water was the solvent used in obtaining these spectra, and a total of 20 electronic states were calculated; however, only the states that reproduced the experimental transitions are presented (Table 4). The absorption spectra were calculated using time-dependent DFT [54] with the same exchange-correlation functional and basis set. All thermochemical calculations were obtained at 298.15 K. The binding energies (Δ*E*) and binding free energies (Δ*G*) of dimers, trimers, and tetramers were calculated as:

Δ*E_binding_* = *E_total_* − ∑*E_monomer_*,*i* (1)

Δ*G_binding_* = *G_total_* − ∑*G_monomer_*,*i* (2)

### 4.7. DC Electrical Conductivity Measurements

DC electrical conductivity measurements were carried out at 25 °C. The resistivities of untreated and treated POMA were measured using a Keithley Model 2612 A from 0.5 to 1.2 V. Powder samples were processed into pellets coated with silver ink on both sides. Electrical contacts were performed in pellets using metal wires and silver paste.

## 5. Conclusions

The HT process of the doped ES-POMA at an elevated temperature (100–200 °C) resulted in the destruction of crystal structure, polymer chain scission, and partial withdrawal of chlorine counter ions. A decrease in some XRD peaks presented in the untreated ES-POMA pattern was observed, resulting in changes to the unit cell parameters due to the decomposition from tetrameric to trimeric-folded chains after the HT process. All these changes influenced the electrical conductivity. The HT process also caused a dedoping of ES-POMA depending upon the treatment temperature. ES-POMA presented well-defined energy levels, showing the typical behavior of a conducting material. After the HT process, some energy levels disappeared due to the ring breaking and partial withdrawal of the chlorine counter ions. The HOMO level of ES-POMA became the highest occupied level of the treated polymer. Thus, this work provides a systematic structural, morphological, and electrical characterization of ES-POMA by combining experimental and theoretical results in the evaluation as a function of temperature.

## Figures and Tables

**Table 1:** Le Bail method performed for ES-POMA, TT_100_, and TT_200_ using the program Fullprof: cell parameters, average size, and anisotropy, crystallite apparent size and agreement factors.

Refined Parameters	ES-POMA	TT_100_	TT_200_
*a* (Å)	7.10275	7.06554	6.99176
*b* (Å)	11.39338	11.42507	10.87043
*c* (Å)	18.82489	15.58464	15.62562
*α* (Å)	82.27626	84.71527	83.43137
*β* (Å)	84.02425	85.33233	84.86502
*γ* (Å)	88.28839	90.64657	88.31005
V (Å^3^)	1501	1248	1175
Average Crystallite Size (anisotr.) (Å)	29 (4)	27 (5)	23 (3)
Crystallite Apparent Size [100] (Å)	37	31	27
Crystallite Apparent Size [010] (Å)	23	20	17
Crystallite Apparent Size [001] (Å)	34	29	26
*R_P_* (%)	3.59	3.92	3.96
*R_WP_* (%)	4.66	4.98	3.73
*X* ^2^	1.13	1.11	1.12

**Table 2:** Bond length of doped-POMA (ES-POMA) tetramer, undoped-POMA tetramer, doped-POMA (ES-POMA) trimer, and undoped-POMA trimer.

Bond Length	Doped-POMA Tetramer (Å)	Undoped-POMA Tetramer (Å)	Undoped-POMA Trimer (Å)	Doped-POMA Trimer (Å)
C–C	1.39	1.40	1.40	1.40
C–N	1.36	1.39	1.41	1.36
C–O	1.39	1.39	1.39	1.39
N–H	1.04	1.00	1.01	1.04
NH–Cl	1.95	-	-	5.91

**Table 3:** Binding energy (Δ*E_binding_*) and Gibbs free energy of binding (Δ*G_binding_*).

Polymers	Δ*E_binding_* (kcal.mol^−1^)	Δ*G_binding_* (kcal.mol^−1^)
Doped (POMA tetramer/POMA trimer)	−186.37	−178.90
Undoped (POMA tetramer/POMA trimer)	−95.61	−77.73
Doped-POMA tetramer/Cl	−88.88	−71.98
Doped-POMA trimer/Cl	6.82	25.42

## Data Availability

The data used to support the findings of this study are available from the corresponding author upon request.

## Sample Availability

Sample of the compounds are not available from the authors.
